# Machine Learning Reveals Ets2 as a Novel Target for Membranous Nephropathy Treatment and Its Role in Immune Infiltration

**DOI:** 10.3389/fmed.2022.813329

**Published:** 2022-03-18

**Authors:** Peng-Zhi Wan, Tian-Hua Xu, Bin-Yao Tian, Guang-Ying Guo, Xiao-Li Li, Li Yao

**Affiliations:** Department of Nephrology, The First Affiliated Hospital of China Medical University, Shenyang, China

**Keywords:** membranous nephropathy, machine learning, immune infiltration, Ets2, chronic kidney disease

## Abstract

**Background:**

Membranous nephropathy (MN) is a common pathological phenotype for adult nephrotic syndrome (NS). The occurrence of MN is increasing across China, but diagnostic methods for MN still rely on kidney biopsy and PLA2R and THSD7A detection in plasma and kidney tissue, and there has been no new biomarker for MN discovered since 2014. Immune infiltration status in MN patients suffers from the dearth of associated studies. In the present study, we aimed to find new bio-markers for MN and evaluate the role of immune cells infiltration in MN pathology.

**Methods:**

We downloaded MN expression profile from the Gene Expression Omnibus database and used R-project to screen differentially expressed genes (DEGs) and performed functional correlation analysis. Least absolute shrinkage and selection operator (LASSO) logistic regression and Radom Forest algorithms were used to screen and verify the bio-markers of MN. Finally, CIBERSORT was used to evaluate the infiltration of immune cells in MN tissues.

**Results:**

A total of 463 DEGs were screened from the MN tissue in this study. ETS2 was identified as bio-marker for MN. The CIBERSORT results showed that there were statistical differences in monocytes, plasma cells, regulatory T cells, and memory B cells. In addition, ETS2 was positively related to monocytes, M1 phase macrophages, and neutrophils and negatively correlated to plasma cells, CD4+ T memory cells, M2 macrophages, CD8+ T cells, memory B cells, and resting mast cells.

**Conclusion:**

(1) Machine learning algorithms reveals Ets2 as a novel target for membranous nephropathy patients. (2) Immune infiltration plays an important part in membranous nephropathy. (3) Ets2 expression is related to immune cells infiltration.

## Introduction

Membranous nephropathy (MN) is one of the common causes of nephrotic syndrome (NS) in the adult population (1). Recent studies confirmed the occurrence of MN in primary glomerulopathy (2). The typical pathological changes for MN include diffuse thickening of the glomerular capillary basement membrane and diffuse granular deposits of IgG with or without C3 (3). Currently, the diagnosis of MN relies on kidney biopsy and detection of PLA2R (4) and THSD7A (5) in plasma and kidney biopsy tissue: there has been no new biomarker for MN discovered since 2014 (6). Finding new biomarkers for MN diagnosis and treatment target is important.

Based on previous studies on human MN patient kidney biopsy and animal models, MN pathophysiological changes involved B cell activation (7) and immune infiltration caused by B cell activation including macrophages (8) and T cells (9). Recently, Rituximab, a monoclonal antibody against CD20, which can cause B cell degeneration, had been approved as a first-line treatment for MN patients (10), indicating that immune infiltration, especially B cell action, plays an important role in MN activation. However, there has been no large-scale immune infiltration study on human MN patients based on RNA sequencing and the CIBERSORT algorithm.

Machine learning algorithms such as least absolute shrinkage and selection operator logistic regression (LASSO) and random forest algorithms were widely used in clinical practice. These machine learning algorithms provided new insight in COVID-19 mortality (11), progressive of idiopathic pulmonary fibrosis (12) and cancer outcomes (13). Based on these studies, we could use machine learning algorithms to find novel targets for MN patients.

In the present study, a microarray dataset of MN downloaded from the Gene Expression Omnibus (GEO) database was used. We performed differential expression gene analysis and used machine learning algorithms to screen and identify suitable targets for MN treatment and diagnosis. CIBERSORT (14) was then used to ascertain the difference in immune infiltration between normal tissue and MN tissue. In addition, MN treatment target and its relationship with immune infiltration in 22 immune cells were determined.

## Materials and Methods

### Data Download

The “GEOquery” package in R (Version 4.10) was used to download the MN expression profile datasets GSE99340 and GSE108113 from the GEO database.

### Data Processing and Deg Screening

The expression matrices of GSE99340 and GSE108113 were downloaded and combined. The control group and MN group samples were selected according to patient data (Table 1). Inter-batch differences were removed using the “sva” package. Two PCA cluster plots were used to demonstrate the effect of inter-sample correction. DEGs were scanned using the “limma” package and “ggplot2” software was used to show differential expression of DEGs.

**Table 1:** Sample number and group information.

**Group**	**GEO accession**	**Sample**
Control group	GSE99340	GSM2641153 GSM2641154 GSM2641155 GSM2641156
Control group	GSE108113	GSM2889865 GSM2889866 GSM2889867 GSM2889868 GSM2889869 GSM2889870 GSM2890047 GSM2890048 GSM2890049 GSM2890050 GSM2890051
MN group	GSE99340	GSM2641237 GSM2641238 GSM2641239 GSM2641240 GSM2641241 GSM2641242 GSM2641243 GSM2641244 GSM2641245 GSM2641246 GSM2641247 GSM2641248 GSM2641249 GSM2641250 GSM2641251 GSM2641252 GSM2641253 GSM2641254 GSM2642375 GSM2642376 GSM2642377 GSM2642378 GSM2642379 GSM2642380 GSM2642381 GSM2642382 GSM2642383 GSM2642384 GSM2642385 GSM2642386 GSM2642387 GSM2642388 GSM2642389 GSM2642390 GSM2642391 GSM2642392 GSM2642393
MN group	GSE108113	GSM2889876 GSM2889877 GSM2889878 GSM2889879 GSM2889880 GSM2889881 GSM2889882 GSM2889883 GSM2889884 GSM2889885 GSM2889886 GSM2889887 GSM2889888 GSM2889889 GSM2889890 GSM2889891 GSM2889892 GSM2889893 GSM2889894 GSM2889895 GSM2889896 GSM2889897 GSM2889923 GSM2889924 GSM2889925 GSM2889926 GSM2889927 GSM2889928 GSM2889929 GSM2889930 GSM2889931 GSM2889932 GSM2889933 GSM2889934 GSM2889935 GSM2889936 GSM2889937 GSM2889938 GSM2889939 GSM2889940 GSM2889941 GSM2889942 GSM2889943 GSM2889944 GSM2890058 GSM2890059 GSM2890060 GSM2890061 GSM2890062 GSM2890063 GSM2890064 GSM2890065 GSM2890066 GSM2890067 GSM2890068 GSM2890069 GSM2890070 GSM2890071 GSM2890072 GSM2890073 GSM2890074 GSM2890075 GSM2890076 GSM2890077 GSM2890078 GSM2890079 GSM2890080 GSM2890081 GSM2890082 GSM2890083 GSM2890084 GSM2890115 GSM2890116 GSM2890117 GSM2890118 GSM2890119 GSM2890120 GSM2890121 GSM2890122 GSM2890123 GSM2890124 GSM2890125 GSM2890126 GSM2890127 GSM2890128 GSM2890129 GSM2890130

### Functional Analysis

The “clusterProfiler” package was used to perform Gene Ontology (GO) and Disease Ontology (DO) enrichment analyses on DEGs, respectively. KEGG pathway enrichment analyses were also conducted on the gene expression matrix through the “clusterProfiler” package. A false discovery rate (FDR) < 0.25 and *p* < 0.05 were considered to represent significant enrichment.

### Screening and Verification of Treatment Target

We used least absolute shrinkage and selection operator logistic regression (LASSO) and random forest algorithms to perform feature selection to screen treatment target for MN. The LASSO algorithm was applied using the “glmnet” package, and the random forest algorithm was established using the “randomForest” package the further to evaluate treatment targets. The GSE108113 matrix was used as a test matrix to examine random forest algorithm results. Then, we combined genes from the results from running the LASSO and random forest algorithms.

### Evaluation of Immune Cell Infiltration

CIBERSORT R-packages and the LM22 document were used to run the algorithm locally (14); we filtered out those samples with *p* < 0.05 and obtained the immune cell infiltration matrix. The “corrplot” package was used to draw a correlation heatmap to visualize the correlation between infiltrated immune cells; the “ggplot2” package was employed to plot diagrams allowing visualization of the correlations in immune cell infiltration.

### Correlation Analysis of Treatment Target and Immune Cell Infiltration

Spearman correlation analysis was applied to the markers and infiltrating immune cells and the “ggplur” package was used to visualize the results.

## Results

### Data Processing and DEGs Screening

**Figure 1:**
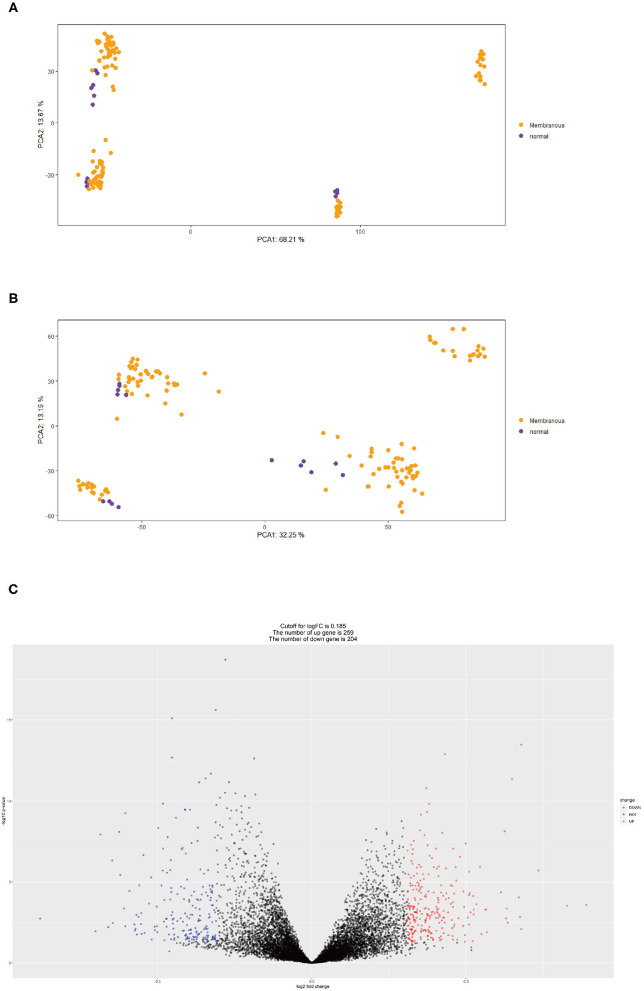
**(A)** PCA clustering results of expression dataset before removing inter-batch difference: yellow dots represent MN samples and purple dots represent normal samples. **(B)** PCA clustering results of expression dataset after removing inter-batch difference, yellow dots represent MN samples and purple dots denote normal samples. **(C)** Volcano map for 463 DEGs, red represents up-regulated DEGs, blue represents down-regulated DEGs.

**Figure 2:**
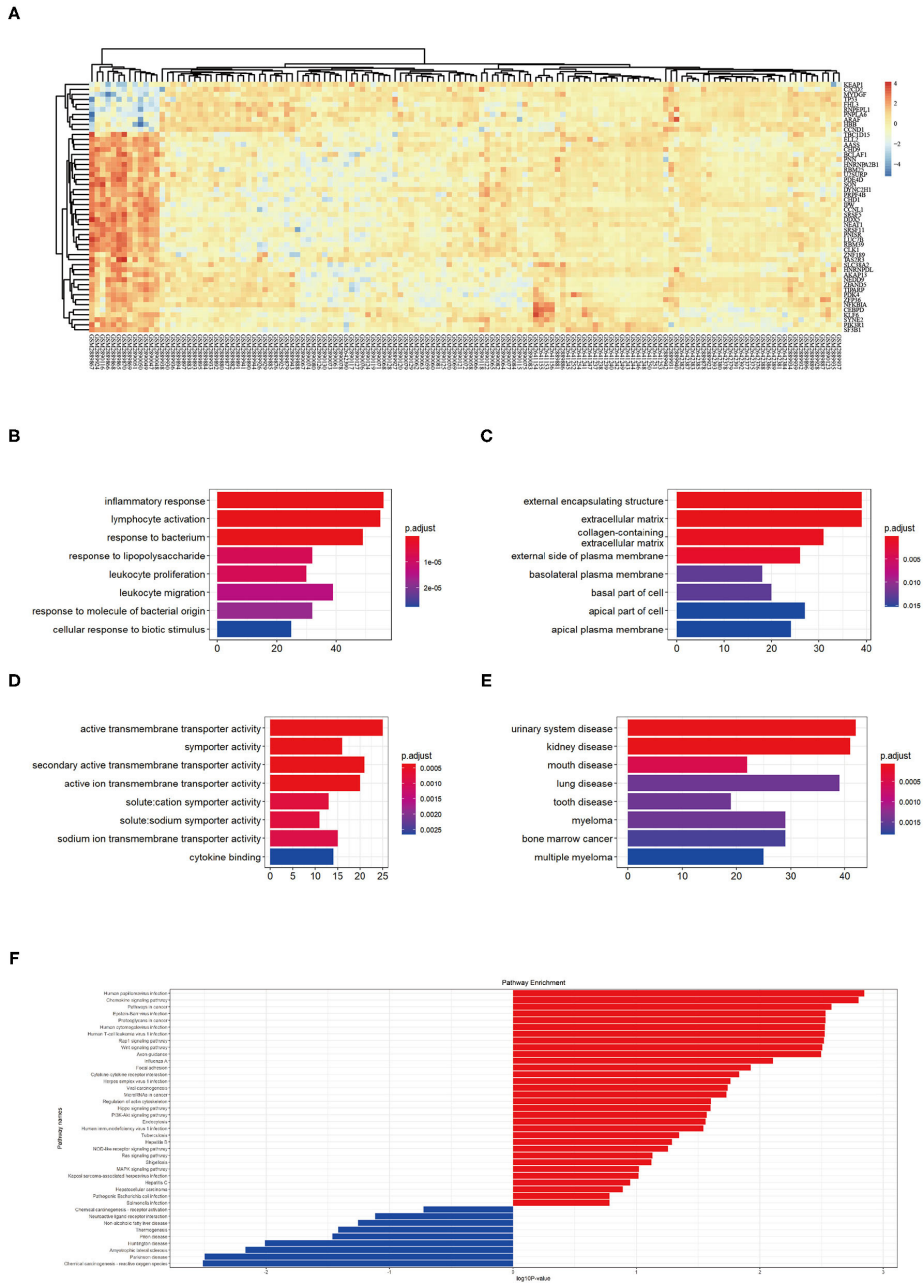
**(A)** Top 50 DEG expressions in each sample, red represents a high level of expression and blue represents a low level of expression. **(B–D)** GO enrichment in biological process (BP), cellular component (CC), and molecular function (MF): color represents the *P*-value, red represents a low *P*-value and blue represents a high *P*-value. **(E)** DO enrichment: color represents the *P*-value, red represents a low *P*-value and blue represents a high *P*-value. **(F)** KEGG pathway enrichment results, red represents up-regulated pathways and blue represents down-regulated pathways.

First, we merged expression profile datasets GSE99340 and GSE108113. The “sva” package was then used to remove inter-batch differences between different expression matrices. The merged expression matrix was normalized and processed: two PCA cluster diagrams (before and after normalization, Figures 1A,B) were used, indicating that the sample source was reliable. After data normalization, R-project was used to extract a total of 463 DEGs from the gene expression matrix, as shown in volcano map (Figure 1C). The heatmap of the top-50 DEGs demonstrated that DEGs were expressed differently in normal and MN samples (Figure 2A).

### Functional Correlation Analysis

GO analysis showed that DEGs were mainly related to biological process (BP) including inflammatory response, lymphocyte activation, response to bacterium, response to lipopolysaccharide, leukocyte proliferation, leukocyte migration, response to molecules of bacterial origin, and cellular response to biotic stimulus (Figure 2B). The cellular component (CC) aspect of the results of GO analysis of DEGs included external encapsulating, extracellular matrix, collagen containing, external side of plasma membrane, basolateral plasma membrane, basal part of cells, and apical plasma membrane (Figure 2C). The molecular function (MF) aspect of the GO analysis results included active transmembrane transporter activity, symporter activity, secondary active transmembrane transporter activity, active ion transmembrane transporter activity, solute cation symporter activity, solute sodium symporter activity, sodium ion transmembrane transporter activity, and cytokine binding (Figure 2D).

GO analysis showed that DEGs were mainly related to urinary system disease, kidney disease, mouth disease, lung disease, tooth disease, myeloma, bone marrow cancer, and multiple myeloma (Figure 2E). KEGG pathway enrichment showed that human papilloma virus infection pathway, chemokine signaling pathway, pathways in cancer, Epstein-Barr virus infection pathway, proteoglycans in cancer pathway, human cytomegalovirus infection pathway, human T-cell leukemia virus 1 infection pathway, Rap 1 signaling pathway, Wnt signaling pathway, and axon guidance pathway as the top-10 most up-regulated pathways (Figure 2F). These results indicating MN pathophysiological changes involved immune responses.

### Screening of Biomarkers for MN

**Figure 3:**
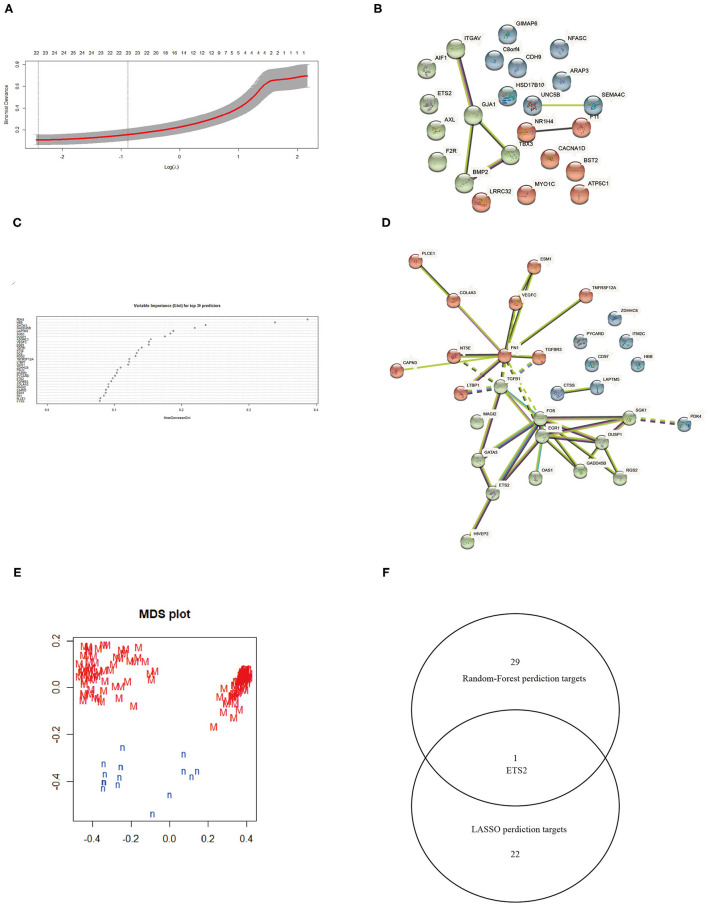
**(A)** Selection of λ in the LASSO algorithm. **(B)** PPI network between LASSO algorithm results. **(C)** Top 30 results from the random forest algorithm. **(D)** PPI network between random forest algorithm results. **(E)** Random Forest algorithm learning status. **(F)** Venn map combining results from the LASSO and random forest algorithms.

The LASSO logistic regression algorithm was employed to identify 23 genes from DEGs as biomarkers for MN combined results from two λ values (Figure 3A). The protein-protein interaction (PPI) was analyzed (Figure 3B); 30 genes were obtained through use of the random forest algorithm as MN biomarkers (Figure 3C) and PPI analysis (Figure 3D); the learning status of the random forest algorithm showed that the predictive results were useful when scanning MN patients (Figure 3E). The gene markers from two algorithms were overlapped and one related gene (ETS2) was obtained (Figure 3F).

### Immune Cell Infiltration Results and Correlation Analysis Between Ets2 and Immune Cells

**Figure 4:**
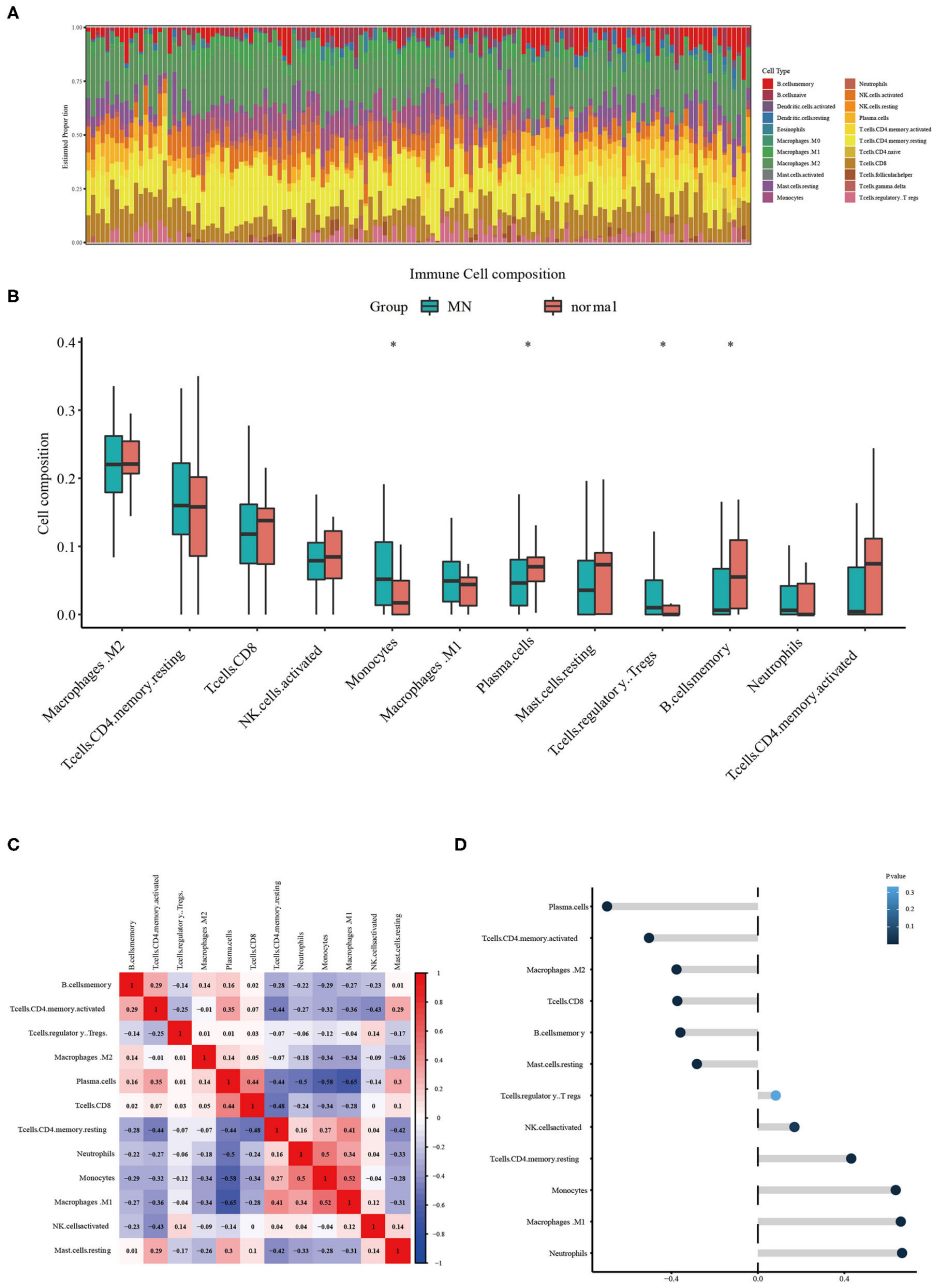
**(A)** Estimated proportion of immune cells in each sample. **(B)** Different immune cell infiltration between MN and normal samples. The symbol * represents a *P*-value < 0.05. **(C)** Correlation between different infiltrated immune cells. **(D)** ETS2 Correlation upon different immune cell infiltrations.

The CIBERSORT tool was used to identify the estimated proportion of immune cells in MN and normal tissues. The estimated proportion of immune cells for each sample is shown in Figure 4A. The immune cell composition in both MN and normal groups was calculated; results showed that there were statistical differences in monocytes, plasma cells, regulatory T cells, and memory B cells (Figure 4B). The correlation between infiltrated immune cells is shown in Figure 4C. The correlation between ETS2 and infiltered immune cells was also determined. ETS2 was positively related to monocytes (cor = 0.63795852, *p* = 3.007799 × 10^−17^), M1 phase macrophages (cor = 0.66141468, *p* = 7.823412 × 10^−19^), and neutrophils (cor = 0.66695409, *p* = 3.149015 × 10^−19^) and negatively correlated to plasma cells (cor = −0.69775865, *p* = 1.369595 × 10^−21^), CD4+ T memory cells (cor = −0.50377651, *p* = 1.119549 × 10^−7^), M2 macrophages (cor = −0.37665222, *p* = 5.751199 × 10^−6^), CD8+ T cells (cor = −0.37258185, *p* = 6.280381 × 10^−6^), memory B cells (cor = −0.35864338, *p* = 1.455759 × 10^−5^), and resting mast cells (cor = −0.28208991, *p* = 7.672348 × 10^−4^) (Figure 4D).

## Discussion

MN is one of the most common pathological phenotypes for adult NS (3). Treatment for MN includes Rituximab, immunosuppressants, and glucocorticoids (10). Although, some patients can self-heal, many suffer from a protracted course of MN and some would eventually progress to end-stage-kidney-disease and require a kidney transplant.

Immune infiltration plays an important role in MN development (15). B cell activation leads to deposition of immune complex causing classic pathological changes of MN. Immune complex and B cell activation also leads to immune cell infiltration by macrophages. Macrophage phenotypes also play a critical role in the progress of MN; MN patients had a higher ratio of M1 macrophages to M2 phenotype, which led to increased inflammation and caused more damage (8).

We downloaded the expression profile dataset from the GEO database and identified a total of 463 DEGs. GO enrichment analysis showed that DEGs were mainly related to inflammatory response, lymphocyte activation, response to bacterium, response to lipopolysaccharide, leukocyte proliferation, leukocyte migration, response to molecules of bacterial origin, and cellular response to biotic stimulus. These results show that immune activity plays an important role in MN processes. KEGG enrichment analysis showed that up-regulated DEGs mainly belonged to the human papilloma virus infection pathway, chemokine signaling pathway, pathways in cancer, and Epstein-Barr virus infection pathway. These results showed that immune infiltration and immunomodulatory genes were changed during MN pathology changes. Also, targeting immune infiltration and virus-related innate immune could be next generation treatments (15) and diagnostic methods for MN (16).

CIBERSORT analysis result of 22 immune cell infiltration showed the statistical differences in monocytes, plasma cells, regulatory T cells, and memory B cells and indicated that pathways involved in immune cell activation and infiltration could be critical in MN progression. Moreover, these results demonstrated that these immune cells could be viable targets for MN immune suppression treatment.

The expression data were analyzed by using machine learning algorithms to screen possible targets for MN treatment. LASSO and random forest algorithms were used during the scan for treatment targets. We then combined the results from the LASSO algorithm and the random forest algorithm, showing that ETS2 was a suitable target for MN treatment. ETS2 was found to be positively related to monocytes, M1 phase macrophages, and neutrophils and negatively correlated to plasma cells, CD4+ T memory cells, M2 macrophages, CD8+ T cells, memory B cells, and resting mast cells. These results indicated that ETS2 participated in MN pathophysiological immune infiltration. According to previous studies, ETS2 participated in various activities: EST2 can control cytokine production and innate immune activation, which leads to IL-6 suppression and decreased amounts of macrophages (17). These functions of ETS2 could be related to its suppression of MAPK/NF-KB pathways (18). Also, deletion of ETS2 in pancreas leads to fibroblast continuous activation (17), however, studies in kidney showed that ETS2 could promote epithelial-to-mesenchymal transition and cause the progression of renal fibrosis. The study also indicated that these results could be caused by direct levels of regulation between ETS2 and JUNB transcription (19). Moreover, some studies showed that ETS2 interacts with the TGF-β/Smad pathway (20)—a pathway related to renal fibrosis—but studies of ETS2 function in MN are needed.

These results demonstrated that ETS2 was a crucial component for MN activation and immune infiltration, but direct intervention with ETS2 transcription function is not a wise way in which to treat MN. Further studies should be aimed at understanding of ETS2 function in MN and interaction with ETS2 promoting JUNB transaction. Inhibiting ETS2 and JUNB promotor binding could be the next-generation treatment used to cure MN. However, there are some limitations of this study, such as lack of the validations of Ets2 in MN patients at the protein level and Ets2 expression level's relation with patients' clinical outcomes. Further studies in this area are needed.

## Data Availability Statement

Publicly available datasets were analyzed in this study. This data can be found here: GEO Database; GSE99340; GSE108113.

## Author Contributions

P-ZW: carried out the basic analysis of the study. T-HX and B-YT: provided clinical study of this work. G-YG: took part in statistical analysis. X-LL and LY: took part in study design. All authors contributed to the article and approved the submitted version.

## Funding

This work was supported by National Natural Science Foundation of China (Project number: 82070763), Key R&D Program of Liaoning Province (Project number: 2020JH 2/10300045 and 2019JH810300016), Population and Health Special Project of Shenyang Science and Technology Plan (Project number: 19-112-4-031), and Servier Kidney Disease Research and Development Project (Project number: 20010040796).

## Conflict of Interest

The authors declare that the research was conducted in the absence of any commercial or financial relationships that could be construed as a potential conflict of interest.

## Publisher's Note

All claims expressed in this article are solely those of the authors and do not necessarily represent those of their affiliated organizations, or those of the publisher, the editors and the reviewers. Any product that may be evaluated in this article, or claim that may be made by its manufacturer, is not guaranteed or endorsed by the publisher.
